# Metabolomic profiling of lung and prostate tumor tissues by capillary electrophoresis time-of-flight mass spectrometry

**DOI:** 10.1007/s11306-012-0452-2

**Published:** 2012-11-02

**Authors:** Kenjiro Kami, Tamaki Fujimori, Hajime Sato, Mutsuko Sato, Hiroyuki Yamamoto, Yoshiaki Ohashi, Naoyuki Sugiyama, Yasushi Ishihama, Hiroko Onozuka, Atsushi Ochiai, Hiroyasu Esumi, Tomoyoshi Soga, Masaru Tomita

**Affiliations:** 1 Institute for Advanced Biosciences, Keio University, Tsuruoka, Yamagata Japan; 2 Systems Biology Program, Graduate School of Media and Governance, Keio University, Fujisawa, Kanagawa Japan; 3 Human Metabolome Technologies, Inc., Tsuruoka, Yamagata Japan; 4 Graduate School of Pharmaceutical Sciences, Kyoto University, Kyoto, Japan; 5 National Cancer Center Hospital East, Kashiwa, Chiba Japan

**Keywords:** Metabolomics, CE-MS, Phosphoproteomics, Lung cancer, Prostate cancer, Tumor microenvironment

## Abstract

Metabolic microenvironment of tumor cells is influenced by oncogenic signaling and tissue-specific metabolic demands, blood supply, and enzyme expression. To elucidate tumor-specific metabolism, we compared the metabolomics of normal and tumor tissues surgically resected pairwise from nine lung and seven prostate cancer patients, using capillary electrophoresis time-of-flight mass spectrometry (CE-TOFMS). Phosphorylation levels of enzymes involved in central carbon metabolism were also quantified. Metabolomic profiles of lung and prostate tissues comprised 114 and 86 metabolites, respectively, and the profiles not only well distinguished tumor from normal tissues, but also squamous cell carcinoma from the other tumor types in lung cancer and poorly differentiated tumors from moderately differentiated tumors in prostate cancer. Concentrations of most amino acids, especially branched-chain amino acids, were significantly higher in tumor tissues, independent of organ type, but of essential amino acids were particularly higher in poorly differentiated than moderately differentiated prostate cancers. Organ-dependent differences were prominent at the levels of glycolytic and tricarboxylic acid cycle intermediates and associated energy status. Significantly high lactate concentrations and elevated activating phosphorylation levels of phosphofructokinase and pyruvate kinase in lung tumors confirmed hyperactive glycolysis. We highlighted the potential of CE-TOFMS-based metabolomics combined with phosphorylated enzyme analysis for understanding tissue-specific tumor microenvironments, which may lead to the development of more effective and specific anticancer therapeutics.

**Electronic supplementary material:**

The online version of this article (doi:10.1007/s11306-012-0452-2) contains supplementary material, which is available to authorized users.

## Introduction

Hyperactivity of glycolysis independent of oxygen availability is a hallmark of cancer metabolism (Warburg effect) (Warburg 1956). Glycolytic energy metabolism of tumor cells is advantageous for perpetual proliferation and meeting the high demand for non-essential amino acids, fatty acids, and nucleotides, although not for efficient production of ATP. Besides glucose, glutamine is significantly consumed by most tumor cells and metabolized to alanine, lactate, and ammonium ions, which are secreted out of the cells, in a process called glutaminolysis (Heiden et al. 2009). Corroborating these features of cancer metabolism, our previous metabolome analyses of colon and stomach tumor tissues, using capillary electrophoresis time-of-flight mass spectrometry (CE-TOFMS), have revealed significantly high tumor concentrations of glycolytic intermediates including lactate, tricarboxylic acid (TCA) cycle intermediates, and amino acids (Hirayama et al. 2009). Moreover, inter-organ metabolomic differences were more significant than normal-versus-tumor differences within the same organ, which revealed the complexity in generalizing a tumor-specific, organ-independent metabolic profile. This suggested that cells alter their metabolism along with tumorigenesis while retaining much of the metabolism that is unique to their organs of origin. To test this hypothesis further and gain an insight into cancer metabolism, we analyzed metabolomic profiles of normal and tumor tissues obtained from lung and prostate cancer patients.

Deciphering the difference in the flow of energy metabolism between cancer and normal cells solely from the tissue metabolome data is often difficult. The activities of most glycolytic enzymes are known to be regulated by phosphorylation; therefore, we used nano-liquid chromatography-tandem mass spectrometry (nanoLC–MS/MS) to quantify phosphorylation levels of 13 sites contained in ten selected enzymes involved in glycolysis and the TCA cycle. The results indicate that tumor metabolomic profile is highly dependent on its organ of origin, and exhibits unique patterns dependent on cancer type as well as differentiation status. This demonstrates the potential of CE-TOFMS-based metabolomics complemented by phosphorylated enzyme analysis for gaining further insight into the complexity and heterogeneity of tumor metabolism.

## Materials and methods

### Sampling and metabolite extraction

All the experiments were conducted according to the study protocol that was approved by the Institution Review Board of the National Cancer Center, Japan. Informed consent was obtained from all the participants.

Tumor and surrounding tissues were surgically resected from nine lung and seven prostate cancer patients, who had been administered with no anticancer drugs or medications that could greatly modify their metabolisms previous to the surgical treatments. Clinical information on the patients is listed in Table 1. The resected tissue samples were immediately frozen in liquid nitrogen and stored at −80 °C until metabolite extraction. Sample tissues were weighed and completely homogenized by multi-beads shocker (Yasuikikai, Osaka, Japan) at 2,000 rpm for 3 min, after adding 0.5 ml ice-cold methanol containing 50 μM methionine sulfone and camphor-10-sulfonic acid as internal standards. The homogenates were mixed with 0.5 ml chloroform and 0.2 ml ice-cold Milli-Q water. After centrifugation at 2,300×*g* for 5 min, the supernatant was centrifugally filtrated through 5-kDa cut-off filters (Millipore, Bedford, MA, USA) at 9,100×*g* for 3 h to remove proteins. The filtrate was centrifugally concentrated in a vacuum evaporator, dissolved with Milli-Q water, and analyzed by CE-TOFMS.

**Table 1:** Clinicopathological information of patients and their tumor tissues. W, M, and P in the differentiation status indicate well-, moderately-, and poorly- differentiated tumors, respectively

Organ	ID	Age	Sex	Type	Stage	Differentiation
Lung	L1	82	Male	Squamous cell carcinoma	2B	M
	L2	82	Male	Squamous cell carcinoma	1B	M
	L3	77	Male	Squamous cell carcinoma	1B	P
	L4	80	Female	Adenocarcinoma	1B	M
	L5	78	Male	Pleomorphic carcinoma	3B	N/A
	L6	81	Male	Adenocarcinoma	1A	W
	L7	56	Male	Squamous cell carcinoma	3B	M–P
	L8	61	Male	Large cell carcinoma	1B	N/A
	L9	57	Male	Adenocarcinoma	1B	P
Prostrate	P1	68	Male	Adenocarcinoma	2	M
	P2	66	Male	Adenocarcinoma	2	P
	P3	67	Male	Adenocarcinoma	2	P
	P4	63	Male	Adenocarcinoma	3	P
	P5	62	Male	Adenocarcinoma	2	M
	P6	65	Male	Adenocarcinoma	2	M
	P7	58	Male	Adenocarcinoma	2	M

### CE-TOFMS analysis and data processing

CE-TOFMS analysis was performed by an Agilent CE system combined with a TOFMS (Agilent Technologies, Palo Alto, CA, USA) as described previously (Ohashi et al. 2008) with slight modifications. Cationic metabolites were separated through a fused silica capillary (50 μm internal diameter × 80 cm total length) preconditioned with a commercial buffer (H3301-1001, Human Metabolome Technologies Inc. (HMT), Tsuruoka, Japan) and filled with 1 M formic acid as electrolyte, and a commercial sheath liquid (H3301-1020, HMT) was delivered at a rate of 10 μl/min. Sample solution was injected at a pressure of 50 mbar for 10 s. The applied voltage was set at 30 kV. Electrospray ionization-mass spectrometry (ESI-MS) was conducted in the positive-ion mode and the capillary and fragmentor voltages were set at 4,000 and 120 V, respectively. Nebulizer pressure was configured at 5 psig and N_2_ was delivered as a drying gas at a rate of 7 l/min at 300 °C. Exact mass data were acquired at the rate of 1.5 cycles/s over a 50–1,000 *m/z* range. Anionic metabolites were analyzed also through the fused silica capillary preconditioned with a commercial buffer (H3302-1022, HMT) and filled with 50 mM ammonium acetate solution (pH 8.5) as electrolyte, and the aforementioned sheath liquid was delivered at a rate of 10 μl/min. Sample solution was injected at a pressure of 50 mbar for 6 s. The nebulizer pressure, drying gas and its flow rate, applied voltage, and scanning condition of the spectrometer were configured in the same manner as the cationic metabolite analysis. ESI-MS was conducted in the negative mode, and the capillary and fragmentor voltages were set at 3,500 and 125 V, respectively. The data obtained by CE-TOFMS analysis were preprocessed using our proprietary automatic integration software, MasterHands. Each metabolite was identified and quantified based on the peak information including *m/z*, migration time, and peak area. The quantified data were then evaluated for statistical significance by Wilcoxon signed-rank test.

### Enrichment of phosphopeptides

Sample tissues were disrupted by multi-beads shocker and suspended in 100 mM Tris–HCl (pH 9.0) containing 8 M urea, protein phosphatase inhibitors and protein phosphatase inhibitors cocktails (Sigma, St. Louis, MO, USA). After centrifugation at 1,500×*g* for 10 min, the supernatant was reduced with 1 mM dithiothreitol, alkylated with 5 mM iodoacetamide, and then digested with Lys-C endopeptidase at 37 °C for 3 h, followed by 5-fold dilution with 50 mM ammonium bicarbonate and digestion with trypsin at 37 °C overnight. The digested samples were desalted using StageTips with SDB-XC Empore disk membranes (3 M, St. Paul, MN, USA) (Rappsilber et al. 2003).

Phosphopeptides were enriched with hydroxy acid-modified metal oxide chromatography (HAMMOC) (Kyono et al. 2008; Sugiyama et al. 2007). Briefly, custom-made metal oxide chromatography tips were prepared using C8-StageTips and titania beads as described previously (Rappsilber et al. 2007). Prior to loading samples, the tips were equilibrated with 0.1 % trifluoroacetic acid (TFA), 80 % acetonitrile and 300 mg/ml lactic acid (solution A). The digested samples from normal or tumor tissues were diluted with 100 μl solution A and loaded into the HAMMOC tips. After successive washing with solution A and solution B (0.1 % TFA and 80 % acetonitrile), 0.5 % piperidine was used for elution. The eluted fraction was acidified with TFA, desalted using SDB-XC-StageTips, and concentrated in a vacuum evaporator, followed by the addition of solution A for subsequent nanoLC–MS/MS analysis. The phosphopeptide enrichment and sample pretreatment was conducted in duplicate.

### NanoLC–MS/MS analysis and database search

NanoLC–MS/MS analyses were conducted using LTQ-Orbitrap (Thermo Fisher Scientific, Rockwell, IL, USA), a Dionex Ultimate 3000 pump (Thermo Fisher Scientific) and an HTC-PAL autosampler (CTC Analytics, Zwingen, Switzerland). A self-pulled needle (150 mm length × 100 μm internal diameter, 6-μm opening) packed with ReproSil C18 materials (3 μm, Dr. Maisch, Ammerbuch, Germany) was used as an analytical column with “stone-arch” frit (Ishihama et al. 2002). A polytetrafluoroethylene-coated column holder (Nikkyo Technos, Tokyo, Japan) was mounted on an *x*–*y*–*z* nanospray interface, and a tee connector with a magnet was used to hold the column needle and to set the appropriate spray position. The injection volume was 5 μl and the flow rate was 500 nl/min for the gradient separation of peptides (Ishihama 2005). The mobile phases consisted of (A) 0.5 % acetic acid and (B) 0.5 % acetic acid and 80 % acetonitrile. A three-step linear gradient of 5–10 % B in 5 min, 10–40 % B in 60 min, 40–100 % B in 5 min and 100 % B in 10 min was used. A spray voltage of 2,400 V was applied via the tee connector. The MS scan range was *m/z* 300–1,500 and the top ten precursor ions were selected for subsequent MS/MS scans. Resolution setting and its maximum injection time were configured at 60,000 and 500 ms, respectively. We also configured the normalized collision energy at 35.0, the isolation width at two, and the minimum signal at 500. Automatic gain controls were set at 500,000 in the MS analysis and at 10,000 in the MS/MS analysis. The capillary temperature was set at 200 °C. A lock mass function was used with a peak derived from polydimethylsiloxane as a lock mass for the LTQ-Orbitrap to obtain constant mass accuracy during gradient analysis (Olsen et al. 2005). Mass Navigator version 1.2 (Mitsui Knowledge Industry, Tokyo, Japan) was used to create peak lists on the basis of the recorded fragmentation spectra. Peptides and proteins were identified by means of automated database searching using Mascot (Matrix Science, London, UK) against UniProt/Swiss-Prot.

## Results and discussion

### Overall metabolomic profile and amino acids

**Fig. 1:**
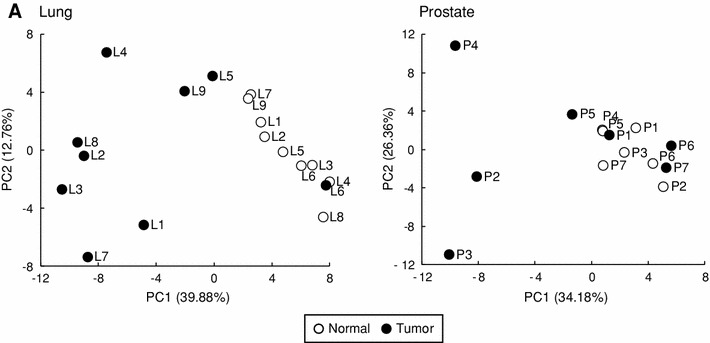

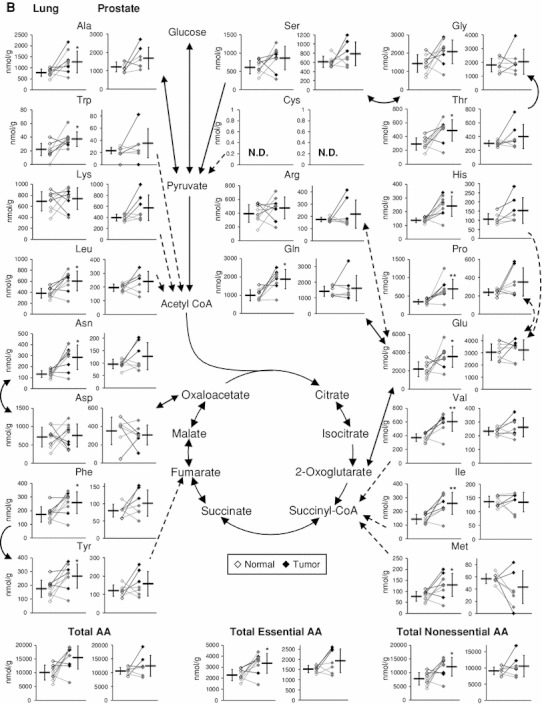
**a** Score plots of PCA using the normalized metabolomic data of paired normal and tumor tissues obtained from lung (*left*) and prostate (*right*) cancer patients. The sample codes correspond to the patient IDs listed in Table 1. *Percentage values* indicated on the axes represent the contribution rate of the first (PC1) and the second (PC2) principal components. **b** Quantified levels of amino acids in normal (*left*, *open dots*) and tumor (*right*, *filled dots*) tissues obtained from lung and prostate cancer patients. *Horizontal bars* represent mean ± SD of normal (*left*) and tumor (*right*) samples and each connected pair represents the values for the same patient. *Gray dots* represent the values for patients with non-SCC lung cancer (L4–L6, L8 and L9) and patients with moderately differentiated prostate cancer (P1 and P5–7). *N.D.* indicates that the metabolite level was below the detection limit of the analysis. *Asterisks* indicate the significant differences between normal and tumor tissue levels based on the Wilcoxon signed-rank test (*p < 0.05; **p < 0.01; and ***p < 0.001)

We analyzed metabolomic profiles of normal and tumor tissues obtained from nine lung and seven prostate cancer patients by using CE-TOFMS. Based on their *m/z* values and migration times, 114 and 86 metabolites were measured in the lung and prostate tissues, respectively (Supplementary Table S1), and visualized on a metabolome-wide pathway map (Supplementary Fig. S1) using VANTED software (Junker et al. 2006). The metabolomic data were then normalized and hierarchically clustered on both the metabolite and sample axes for a heat map representation (Supplementary Fig. S2) and further analyzed by principal component analysis (PCA) using MeV software (Saeed et al. 2003). Thirty-nine metabolites including glycolytic and TCA cycle intermediates, amino acids, and purine nucleoside phosphates, were absolutely quantified (Supplementary Table S2). PCA indicated that tumor metabolomic profiles were much more heterogeneous than their normal counterparts and comprised multiple clusters (Fig. 1a). With reference to the patient information (Table 1) and the hierarchically-clustered samples (Supplementary Fig. S2), tumor types appeared to play a greater part than tumor stage or differentiation status in altering the overall metabolomic profile in lung cancer, whereas differentiation status contributed more in prostate cancer. Indeed, the cluster of squamous cell carcinoma (SCC) patients (L1–3 and L7) was well-distinguished from that of adenocarcinoma (L4, L6 and L9) and pleomorphic carcinoma (L5). This may reflect the intrinsic pathological difference that adenocarcinoma cells but not squamous carcinoma cells retain their function of secreting mucus as glandular epithelial cells. In prostate samples, poorly differentiated prostate tumors (P2–4) were distant from the cluster of moderately differentiated (P1 and P5–7) tumors, which overlapped with that of normal samples. This may be due to higher duct-forming capacity and hormone response of well-differentiated prostate tumors, as well as normal prostate cells, than that of poorly differentiated tumors.

Both lung and prostate tumor samples were well-separated primarily along the PC1 axis; thus, factor loadings for the PC1 axis were evaluated. Correlations with the PC1 were particularly high in branched-chain amino acids (BCAAs) such as Val (*R* = −0.97), Ile (−0.97), and Leu (−0.89) in lung, and Leu (−0.87) in prostate samples (Supplementary Fig. S3). BCAAs are known to be avidly taken up by tumors and highly oxidized in cancer patients (Baracos and Mackenzie 2006), and thus may serve as effective indicators for diagnosing lung tumors. In fact, average lung tumor concentrations of all the 19 amino acids measured were higher than their respective normal levels, as were average prostate tumor levels of all the amino acids except Asp, Ile and Met (Fig. 1b). This is possibly due to hyperactivity of protein degradation and amino acid transporters in tumor cells (Fuchs and Bode 2005; Vander Heiden et al. 2009). Although average tumor levels of most amino acids in lung samples were significantly higher than their respective normal levels, normal and tumor Asp levels were comparable. Asp may be actively consumed as a precursor for nucleic acids and these TCA cycle intermediates, because tumor concentrations of malate, fumarate and succinate were significantly higher than the normal levels. In prostate tissues, levels of some amino acids such as Asn, Lys, Phe, Ser and Tyr and total essential amino acids were particularly higher in poorly differentiated tumors (P2–4; black in Fig. 1b) than moderately differentiated tumors (P1 and P5–7; gray in Fig. 1b), of which levels were comparable to the corresponding normal levels (Fig. 1b), implying enhancement of acquiring the amino acids upon dedifferentiation of prostate cancer cells.

### Energy charge and adenosine and guanosine phosphates

**Fig. 2:**
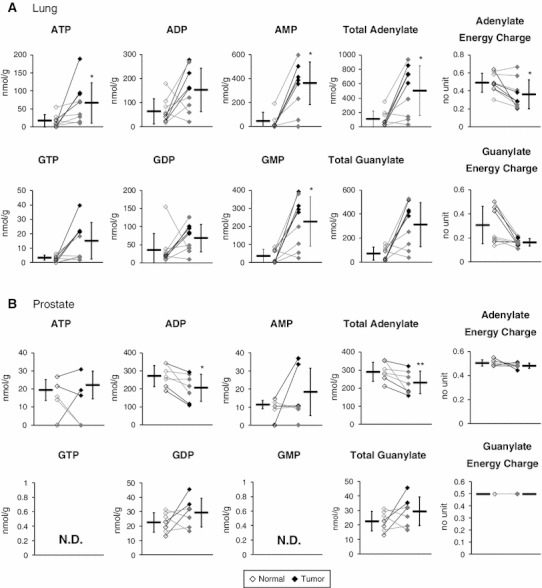
Adenosine and guanosine phosphates, total adenylates and guanylates, and adenylate and guanylate energy charges of normal (*left*, *open dots*) and tumor (*right*, *filled dots*) tissues obtained from lung (**a**) and prostate (**b**) cancer patients. *Horizontal bars* represent mean ± SD of normal (*left*) and tumor (*right*) samples and each connected pair represents the values for the same patient. *Gray dots* represent the values for patients with non-SCC lung cancer (L4–L6, L8 and L9) and patients with moderately differentiated prostate cancer (P1 and P5–7). *N.D.* indicates that the metabolite level was below the detection limit of the analysis. *Asterisks* indicate the significant differences between normal and tumor tissue levels based on the Wilcoxon signed-rank test (*p < 0.05; **p < 0.01; and ***p < 0.001)

Adenylate and guanylate energy charges (([RTP] + 0.5 × [RDP])/([RTP] + [RDP] + [RMP]), R = A or G) were lower in lung tumor than normal tissues (Fig. 2a); however, tumor levels were significantly higher than normal levels for ATP (3.8-fold), GTP (4.2-fold), and the other adenosine and guanosine phosphates (1.9–7.9-fold), and hence total adenylates (4.5-fold) and guanylates (4.0-fold). Tumor concentrations of these metabolites were relatively higher in all the SCC samples (L1–3 and L7; black in Fig. 2a) than the others (gray in Fig. 2a) such as L5 and L6, whose overall tumor metabolomic profiles resembled their respective normal profiles (Fig. 1). Purine synthesis may thus be hyperactivated in lung tumors, especially SCC, probably with a high basal ATP ↔ ADP turnover and purine salvage for maximizing their growth. Although prostate tissues showed much less normal-versus-tumor differences, tumor ADP level was significantly lower than normal level (Fig. 2b). High absolute concentrations of ADP and GDP among other purine nucleoside phosphates are unique to prostate tissues, and ATP and AMP levels were relatively higher in poorly differentiated tumors (P2–4; black in Fig. 2b) than moderately differentiated tumors (P1 and P5–7; gray in Fig. 2b). This might be due to a differential expression of adenylate kinase catalyzing the reaction, 2ADP ↔ ATP + AMP, which is undetectable in adult prostate but shows activity along with its malignant alteration (Hall et al. 1985).

### Glycolytic and TCA cycle intermediates and phosphorylated enzymes

**Fig. 3:**
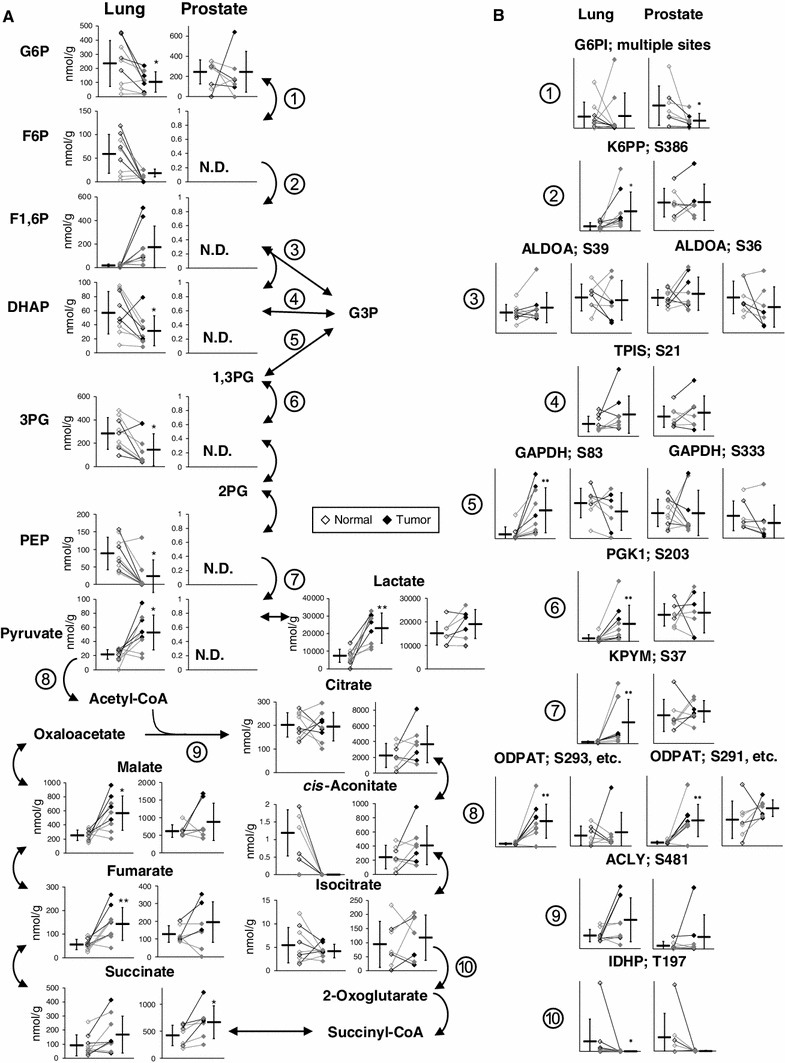
Quantified levels of glycolytic and TCA cycle intermediates (**a**) and phosphorylation levels of each phosphorylation site in associated enzymes (**b**) in normal (*left*, *open dots*) and tumor (*right*, *filled dots*) tissues obtained from lung and prostate cancer patients. *Encircled numbers* in (**a**) indicated next to the metabolic reactions involved in glycolysis and the TCA cycle correspond to the associated enzymes in (**b**). *Horizontal bars* represent mean ± SD of normal (*left*) and tumor (*right*) samples and each connected pair represents the values for the same patient. *Gray dots* represent the values for patients with non-SCC lung cancer (L4–L6, L8 and L9) and patients with moderately differentiated prostate cancer (P1 and P5–7). *N.D.* indicates that the metabolite level was below the detection limit of the analysis. *Asterisks* indicate the significant differences between normal and tumor tissue levels based on the Wilcoxon signed-rank test (*p < 0.05; **p < 0.01; and ***p < 0.001). *G6PI* glucose 6-phosphate isomerase; *K6PP* 6-phosphofructokinase; *ALDOA* aldolase A; *TPIS* triosephosphate isomerase; *GAPDH* glyceraldehydes 3-phosphate dehydrogenase; *PGK1* phosphoglycerate kinase 1; *KPYM* pyruvate kinase isozymes M1/M2; *ODPAT* pyruvate dehydrogenase E1 component subunit alpha; *ACLY* ATP-citrate synthase; and *IDHP* isocitrate dehydrogenase

Most glycolytic and TCA cycle intermediates were absolutely quantified (Fig. 3a), and phosphorylation levels of associated enzymes were also examined (Fig. 3b). Tumor lactate levels were higher than normal levels in both lung and prostate tissues, indicating their enhanced glycolysis and lactate fermentation, which reaffirmed the Warburg effect in cancer. Lung tumor levels of fructose 6-phosphate and fructose 1,6-bisphosphate were significantly lower and higher, respectively, than their corresponding normal levels. This may be partly explained by significantly high tumor levels of S386 phosphorylation in phosphofructokinase, which enhances its activity (Brand and Soling 1975), and thus the overall glycolytic flux because it is a bottleneck enzyme. Although tumor levels of S83 phosphorylation in glyceraldehyde 3-phosphate dehydrogenase and S203 in phosphoglycerate kinase-1 were significantly higher than their respective normal levels, their functional impacts are unknown. Tumor level of S37 phosphorylation of pyruvate kinase, which enhances its activity (Le Mellay et al. 2002), was significantly higher than the normal level. This may rationalize the trend that phosphoenolpyruvate and pyruvate were significantly lower and higher, respectively, in tumor than normal tissues. Tumor levels of S293 and S291 phosphorylation in pyruvate dehydrogenase, which inhibit its activity (Korotchkina and Patel 2001; Patel and Korotchkina 2001), were significantly higher than normal levels in all the lung cancer patients, except L6. This inhibition may contribute to the enhanced glycolysis and resulting lactate accumulation in lung tumors. In prostate tissues, however, most glycolytic intermediates were not detected, probably owing to inevitable over-dilution of the samples for reducing polyamine concentrations, which otherwise adversely interfere with CE-TOFMS analysis. Trivial differences were observed between normal and tumor prostate phosphorylation levels of most glycolytic enzymes except glucose 6-phosphate isomerase (G6PI); nevertheless, the impact of elevated phosphorylation on the activity of G6PI is uncertain. Although intriguing, there was no apparent correlation between significantly high tumor levels of S481 phosphorylation in ATP citrate lyase in SCC samples, L1, L3 and L7, and their citrate concentrations. The impact of elevated phosphorylation levels of T197 in isocitrate dehydrogenase in normal L2 and P2 samples was also unclear. We need a larger number of sample sets in order to validate these results and provide further insight into possible correlations between phosphorylated states of the enzymes and metabolomic profiles.

Levels of all the quantified TCA cycle intermediates were higher in tumor than normal prostate tissues (Fig. 3a), which may be related to the typically hypoxic microenvironment of prostate tissues, because most TCA cycle intermediates are known to increase under hypoxia, while their flux through the pathway remains low (Wiebe et al. 2008). Average prostate citrate concentrations were >11-fold higher than in lung. This was partly due to a high concentration of zinc in the prostate, which inhibits m-aconitase and results in citrate accumulation (Mycielska et al. 2009). Prostate tumor exhibits low zinc levels and elevated fatty acid synthesis consuming citrate, and thus its citrate level is typically lower than in normal tissues (Mycielska et al. 2009), which is, however, inconsistent with our results. Tumor citrate, *cis*-aconitate, and isocitrate levels in P1, P3 and P7 were consistently lower than their respective normal levels, leaving the possibility that zinc and m-aconitase activity levels may vary depending on a factor other than differentiation status.

Succinate, fumarate, and malate levels were markedly higher in both prostate and lung tumor tissues than their corresponding normal tissues (Fig. 3a), which was consistent with our previous results for colon and stomach tumor metabolomics (Hirayama et al. 2009). We recently obtained strong evidence that, especially under hypoxic and nutrient-deprived conditions, energy generation of cancer relies on fumarate respiration (Sakai et al. 2012; Tomitsuka et al. 2010). This confers upon cells the ability to produce ATP by harnessing fumarate to succinate conversion, rather than oxygen to water, as the final electron transport step via the reverse reaction of succinate dehydrogenase (Kita and Takamiya 2002). Accumulation of these metabolites in tumors may therefore be attributed to hyperactivity of fumarate respiration.

### Tumor-specificity and organ-dependency in metabolic profiles

The metabolome data obtained from both lung and prostate tissues were collectively normalized and hierarchically clustered (Supplementary Fig. S4A). As a result, lung-versus-prostate differences in terms of the overall metabolomic profiles appeared to be more significant than normal-versus-tumor differences within the same organ, as observed in our previous comparative metabolome analyses in colon and stomach tissues (Hirayama et al. 2009). As expected, PCA with the collectively normalized data showed clear inter-organ differences along with the PC2 axis; however, the normal-versus-tumor distinctions were also observed along with the PC1 axis (Supplementary Fig. S4B). This suggests that, in the carcinogenic process, cells alter their metabolism with a certain ‘metabolic directionality’ that is independent of organ types while retaining much of the metabolism that is unique to their organs of origin. Metabolites that showed high correlations with the PC2 included several nucleosides, TCA cycle intermediates, and polyamines, which characterize the inter-organ metabolic differences (Supplementary Table S3). In contrast, most glucogenic amino acids such as Thr, Ile, Asn, Pro, His, Gln, and Ser were closely associated with the PC1 (Supplementary Table S3), suggesting that a hyper-production and/or -acquisition of a certain set of amino acids likely occurs in the course of tumorigenesis.

## Conclusion

Overall tumor metabolomic profiles were found to be significantly different depending on tumor type in lung cancer and differentiation status in prostate cancer. Elevated tumor concentrations of almost all the amino acids, especially BCAAs, were identified in an organ-independent manner, and this trend was more prominent in SCC than the other tumor types in lung cancer and in poorly rather than moderately differentiated prostate cancer. Analyses with much more samples, however, are necessary in order to statistically confirm these unique subtype-specific metabolic fingerprint of cancer. In contrast, through our combined metabolomic and phosphorylated enzyme analyses, we found that glycolytic and TCA cycle intermediates, levels of which are probably associated with enzyme phosphorylation levels, exhibited significant organ dependency, reaffirming that inter-organ metabolomic differences are generally more significant than normal-versus-tumor differences within the same organ. Nonetheless, metabolomic profiles of both lung and prostate tumors appear to have a common ‘directionality’ along with their increasing malignancy represented by high concentrations of a certain set of glucogenic amino acids. Taken together, we identified organ-dependent, tumor-specific, and tumor-pathology-dependent metabolic features, which highlights the need for a combined metabolomics and phosphoproteomics analysis on a broader scale with a larger number of sample sets for improving specificity and effectiveness of personalized anticancer therapeutics.

## Electronic supplementary material

Below is the link to the electronic supplementary material.

Supplementary material 1 (XLS 143 kb)

Supplementary material 2 (PNG 3708 kb)

Supplementary material 3 (DOC 29 kb)

Supplementary material 4 (PPT 785 kb)
