# Supplementary material for: Metabolomic profiling of lung and prostate tumor tissues by capillary electrophoresis time-of-flight mass spectrometry
Source: Metabolomics. 2012 Nov 2;9(2):444–53. doi: 10.1007/s11306-012-0452-2 (PMC3608864; doi:10.1007/s11306-012-0452-2)
Supplement: Supplementary file 3 — Supplementary material 3 (DOC 29 kb) [file 11306_2012_452_MOESM3_ESM.doc]

**Supplementary Figure Legends**

Supplementary Fig. S1. Average levels of all the analyzed metabolites in normal (blue bars) and tumor (red bars) tissues obtained from lung (left) and prostate (right) cancer patients. The results show the means ± SD. Asterisks indicate the significant differences between normal and tumor tissue levels based on the Wilcoxon signed-rank test (*, *p* < 0.05; **, *p* < 0.01; and ***, *p* < 0.001).

Supplementary Fig. S2. Heat map representation of all the analyzed metabolites in normal (L1N–L9N) and tumor (L1T–L9T) tissues obtained from lung cancer patients (**A**) and normal (P1N–P7N) and tumor (P1T–P7T) prostate tissues obtained from prostate cancer patients (**B**). Cells in gray indicate that the metabolite was not detected. The metabolome data obtained by CE-TOFMS were preliminarily normalized by evaluating z-values and both the sample and metabolite axes were hierarchically clustered based on the Pearson correlation coefficients.

Supplementary Fig. S3. Normal (left, open dots) and tumor (right, filled dots) levels of metabolites that showed high correlations with PC1 (10 metabolites from the top). Horizontal bars represent mean ± SD of normal (left) and tumor (right) samples and each connected pair represents the values for the same subject. Gray dots represents the values of non-SCC (L4–L6, L8, and L9) patients in lung cancer and of patients with moderately differentiated tumors (P1 and P5–7) in prostate cancer.

Supplementary Fig. S4. **A** Heat map representation of all the analyzed metabolites in normal (L1N–L9N) and tumor (L1T–L9T) tissues obtained from lung cancer patients and normal (P1N–P7N) and tumor (P1T–P7T) prostate tissues obtained from prostate cancer patients. Cells in gray indicate that the metabolite was not detected. The metabolome data of both the lung and prostate tissues obtained by CE-TOFMS were collectively normalized by evaluating z-values and then both the sample and metabolite axes were hierarchically clustered based on the Pearson correlation coefficients. **B** Score plots of PCA using the collectively normalized metabolomic data of paired normal and tumor tissues obtained from lung and prostate cancer patients. The sample codes correspond to the patient IDs listed in Table 1. Percentage values indicated on the axes represent the contribution rate of the first (PC1) and the second (PC2) principal components.
