# Supplementary material for: Metabolomic profiling of lung and prostate tumor tissues by capillary electrophoresis time-of-flight mass spectrometry
Source: Metabolomics. 2012 Nov 2;9(2):444–53. doi: 10.1007/s11306-012-0452-2 (PMC3608864; doi:10.1007/s11306-012-0452-2)
Supplement: Supplementary file 4 — Supplementary material 4 (PPT 785 kb) [file 11306_2012_452_MOESM4_ESM.ppt]

## Slide 1
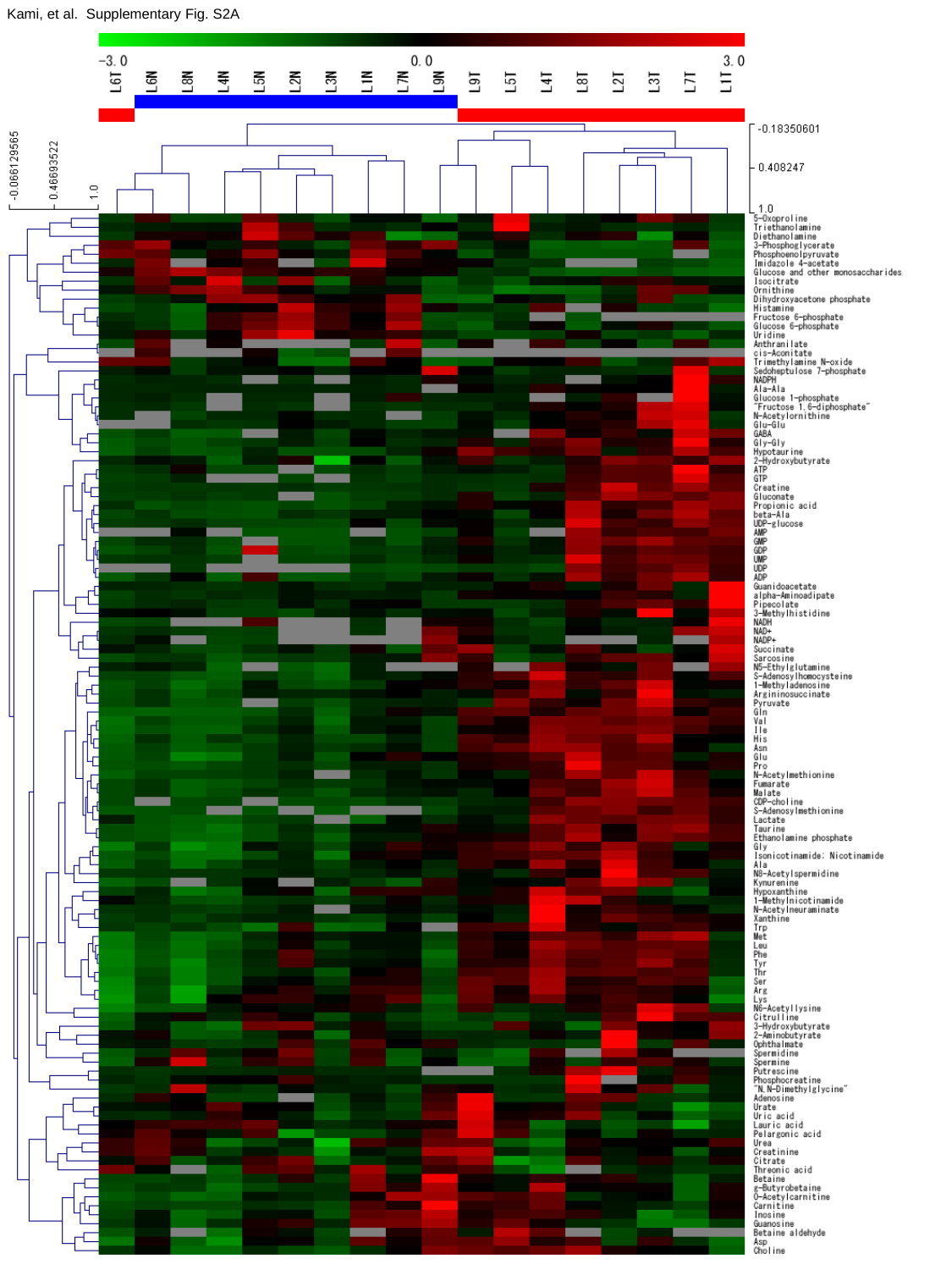

Kami, et al. Supplementary Fig. S2A

## Slide 2
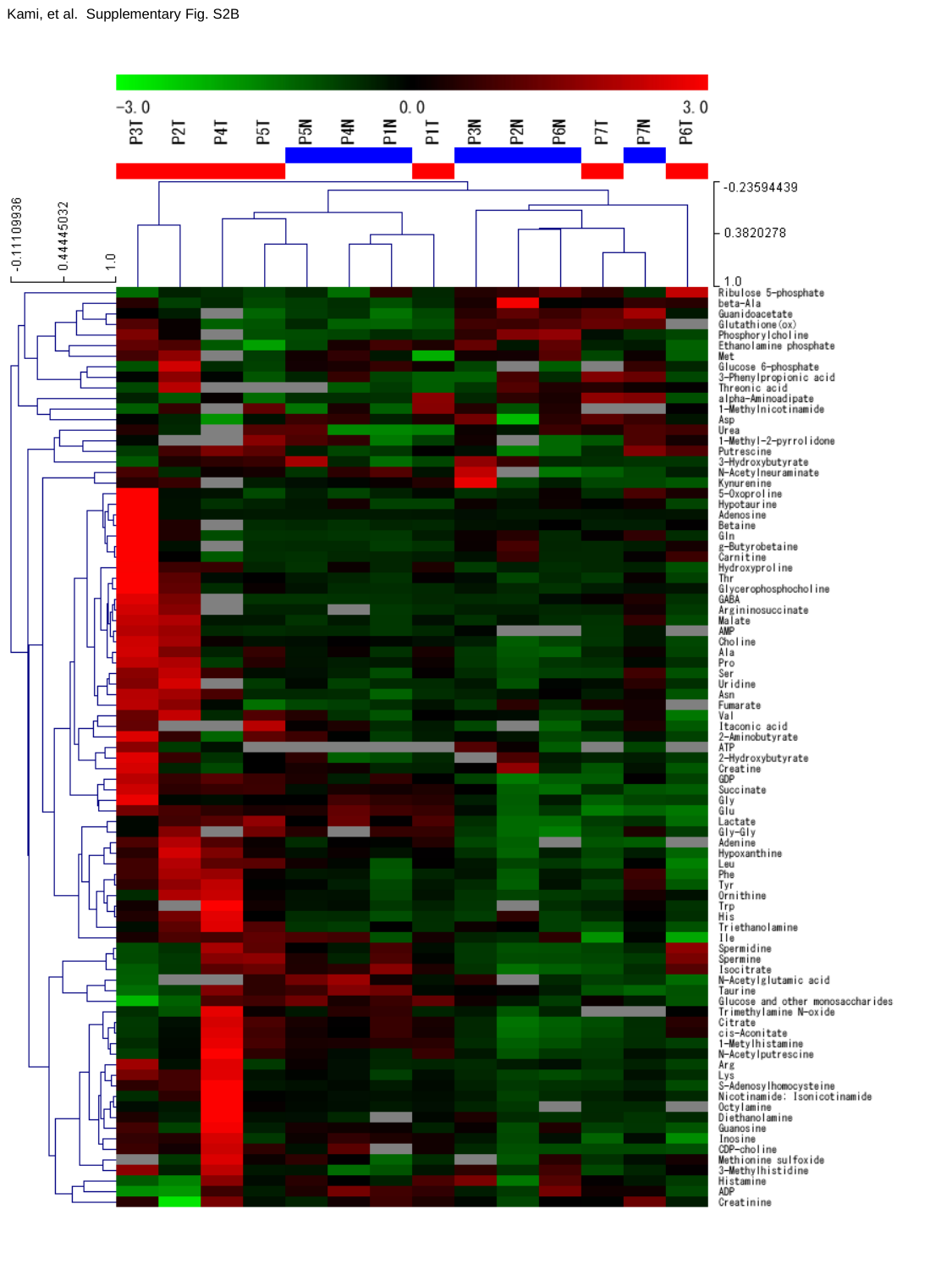

Kami, et al. Supplementary Fig. S2B

## Slide 3
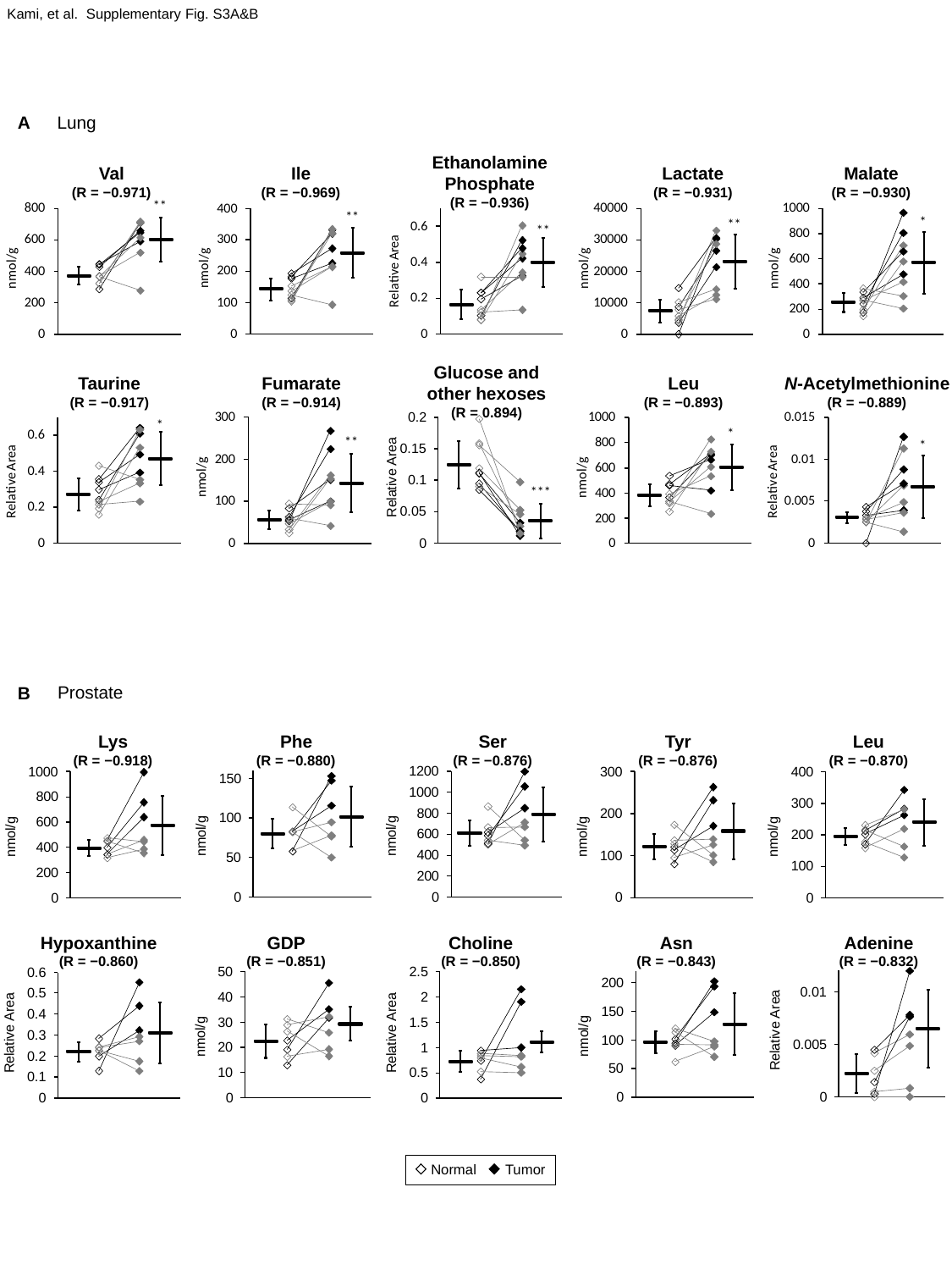

Kami, et al. Supplementary Fig. S3A&B
A
Lung
Ethanolamine
Phosphate
(R = −0.936)
Val
(R = −0.971)
Ile
(R = −0.969)
Lactate
(R = −0.931)
Malate
(R = −0.930)
Glucose and
other hexoses
(R = 0.894)
Taurine
(R = −0.917)
Fumarate
(R = −0.914)
Leu
(R = −0.893)
N-Acetylmethionine
(R = −0.889)
Prostate
B
Lys
(R = −0.918)
Phe
(R = −0.880)
Ser
(R = −0.876)
Tyr
(R = −0.876)
Leu
(R = −0.870)
Hypoxanthine
(R = −0.860)
GDP
(R = −0.851)
Choline
(R = −0.850)
Asn
(R = −0.843)
Adenine
(R = −0.832)
Normal
Tumor

## Slide 4
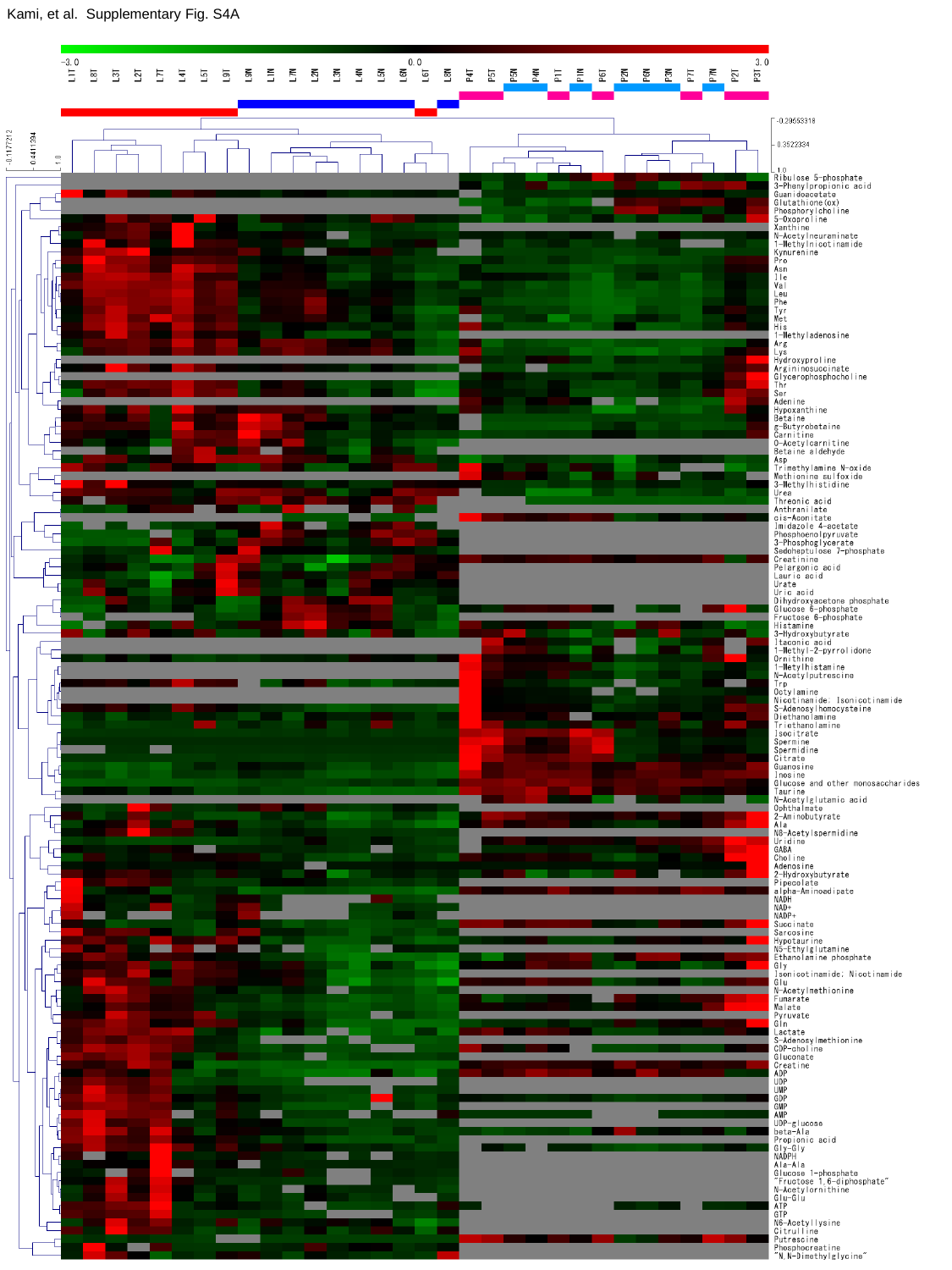

Kami, et al. Supplementary Fig. S4A

## Slide 5
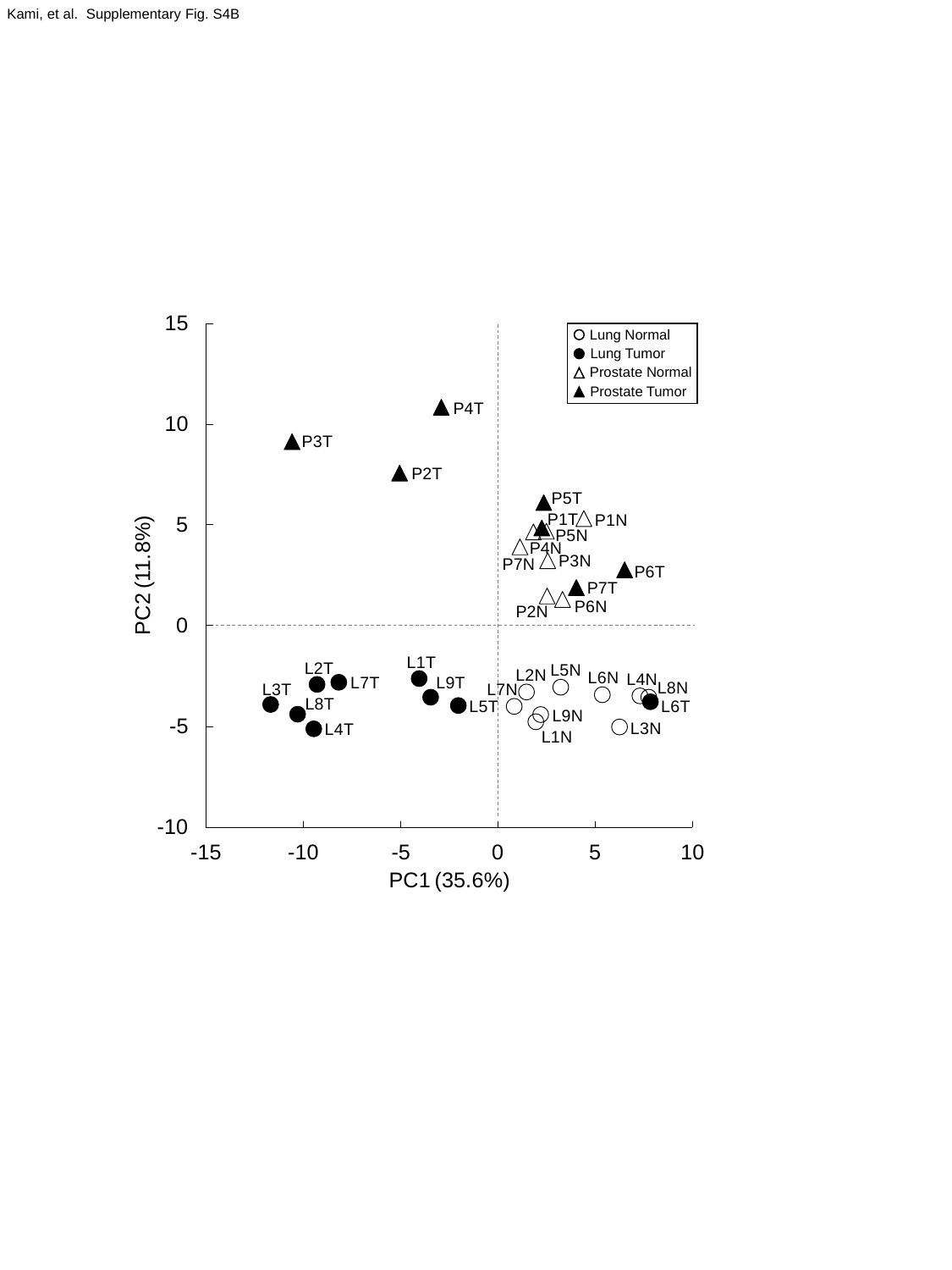

Kami, et al. Supplementary Fig. S4B
Lung Normal
Lung Tumor
Prostate Normal
Prostate Tumor
